# Spring–damper equivalents of the fractional, poroelastic, and poroviscoelastic models for elastography

**DOI:** 10.1002/nbm.3854

**Published:** 2017-11-27

**Authors:** Sverre Holm

**Affiliations:** ^1^ Department of Informatics University of Oslo Oslo Norway

**Keywords:** fractional viscoelasticity, MR elastography (MRE), poroelasticity

## Abstract

In MR elastography, it is common to use an elastic model for the tissue's response in order to interpret the results properly. More complex models, such as viscoelastic, fractional viscoelastic, poroelastic, or poroviscoelastic ones, are also used. These models appear at first sight to be very different, but here it is shown that they may all be expressed in terms of elementary viscoelastic models.

For a medium expressed with fractional models, many elementary spring–damper combinations are added, each of them weighted according to a long‐tailed distribution of time constants or relaxation frequencies. This may open up a more physical interpretation of fractional models.

The shear‐wave component of the poroelastic model is shown to be modeled exactly by a three‐component Zener model. The extended poroviscoelastic model is found to be equivalent to what is called a non‐standard four‐parameter model. Accordingly, the large number of parameters in the porous models can be reduced to the same number as in their viscoelastic equivalents. While the individual displacements from the solid and fluid parts cannot be measured individually, the main use of the poro(visco)elastic models is therefore as a physics‐based method for determining parameters in a viscoelastic model.

## 1 INTRODUCTION

In modeling of data from MR elastography, it is common to use a simple elastic model for the medium. This is the case even for ultrasound elastography. For more accurate modeling, three families of models are used: these are the linear viscoelastic models, such as the Kelvin–Voigt and Zener models, the fractional extensions of these models, and poroelastic models based on the theory of Biot.

The linear viscoelastic models are among those that have been used for fitting the frequency dependence of shear‐wave data from MR elastography in the brain.^1^ The fractional Kelvin–Voigt model was fitted to breast MR elastography data by Sinkus et al^2^ and also analyzed by Holm and Sinkus^3^ and compared with other models for elastography by Zhang and Holm.^4^

It may be argued that these single‐phase models are too simplistic and that in tissue a biphasic model, which distinguishes between solid and liquid phases, would be more accurate. This potential has already been demonstrated in models of the quasi‐static biomechanics of hydrocephalus 5 and infusion‐induced swelling in the brain.^6^ The poroelastic model has also been used for elastography. In the work of Konofagou et al,^7^ ultrasound elastography was modeled with either an elastic model (i.e. without viscosity) or a simplified poroelastic model, which depended on porosity, composite density, and fluid density. Similarly, Perriñez et al^8^ evaluated the full poroelastic model versus an elastic one for MR elastography. McGarry et al^9^ went one step further and compared the poroleastic model with a viscoelastic one with a complex shear modulus. They also tried to use these models for inversion and observed that the viscoelastic model produced better reconstructions at 50 Hz, while the poroelastic model was superior at 1 Hz.

The challenge in making a reconstruction algorithm based on the poroelastic model is the need to capture the displacement of the solid and the fluid independently. Chapter 5 of Hirsch et al^10^ states that, due to the voxel size of MR elastography, it cannot detect the properties of individual pores but only sees a homogeneous effective medium. Further, since it is sensitive to signals from hydrogen atoms in the voxel, one cannot separate the signal from the solid and the fluid, even if the solid should contain fluid that is considered not to be in the pores. Likewise, ultrasound is scattered from the soft tissue, which is mainly part of the solid matrix. Ultrasound will therefore produce a strain estimate in the solid matrix that is only indirectly influenced by the wave in the fluid.^7^ In this article, however, the goal is not to develop a poroelastic model for reconstruction of the MR elastography image but only to discuss it in the framework of explaining variations in parameters due to physiological and physical parameters.

The claim of this article is that these models are much more similar than they appear to be. The fractional models can be developed as sums of ordinary viscoelastic elements weighted in a particular way. The fractional model has its strength, in that it gives a parsimonious description of the phenomenon, i.e. one with a minimal number of parameters, especially when power‐law variation in frequency is observed, but it is not fundamentally different. Likewise, the poroelastic model for the wave mode of interest for elastography, the shear wave, can be described in terms of standard viscoelastic models. In this case, it is the viscoelastic model that requires the smallest number of parameters. The strength of the poroelastic formulation is that it provides a way to find out how these parameters depend on physical and even physiological parameters.

The linear viscoelastic model is therefore first generalized in Section 2 from simple two‐ and three‐component models to chains of spring–damper elements described by time‐ and frequency‐spectral functions. These functions are used to show that the fractional viscoelastic models in Section 3 can be described as sums of ordinary viscoelastic models. A surprising result is that the weighting in the sum follows a long‐tailed distribution, reminiscent of that produced by a fractal geometry.

The shear‐wave solution of the poroelastic theory is then developed in Section 4, from both the original formulation of Biot^11^ and also that of Stoll.^12^ Somewhat unexpectedly, it is found that there is a one‐to‐one correspondence between the poroelastic shear‐wave response and that of a simple spring–dashpot network.

## 2 LINEAR VISCOELASTIC MODELS

The linear viscoelastic model is expressed in several different ways. These ways are needed in order to generalize from simple two‐ and three‐component models (e.g. Kelvin–Voigt and Zener) to higher order models.

In order to illustrate the similarities between models, it is sufficient here to express the models in one dimension, although three dimensions are really needed for a complete shear‐wave description. This section, as well as Section 3, builds to a large degree on the work of Mainardi.^13^

### 2.1 Three descriptions

In linear viscoelasticity, there are three different ways of describing the medium's response. The first is the hereditary model of Boltzmann,(14, 15) where the constitutive equation is a convolution integral^13^:

(1) $$\sigma (t)=G(t)*\frac{\partial \epsilon (t)}{\partial t}$$

The kernel *G*(*t*) is called the relaxation modulus and represents a fading memory, i.e. one where changes in the past have less effect now than more recent changes. In order to ensure causality, the kernel, *G*(*t*), has to be zero for a non‐negative time. The relaxation modulus is the strain response of the system to a step excitation in strain.

The second description is a linear differential equation between stress and strain with constant coefficients:

(2) $$\left[1+\sum_{k=1}^{p}a_{k}\frac{\partial^{k}}{\partial t^{k}}\right]\sigma (t)=\left[E_{e}+\sum_{k=1}^{q}b_{k}\frac{\partial^{k}}{\partial t^{k}}\right]\epsilon (t).$$

The third description is in the form of a relaxation spectrum or Prony expansion (Mainardi's Equation 2.28)^13^:

(3) $$\begin{array}{ccc} G(t) & =G_{e}+G_{\tau }+G_{-}\delta (t) & \\ G_{\tau }(t) & =\sum_{n=1}^{N-1}E_{n}\exp(-t/\tau_{n}) \end{array}$$

**Figure 1:**
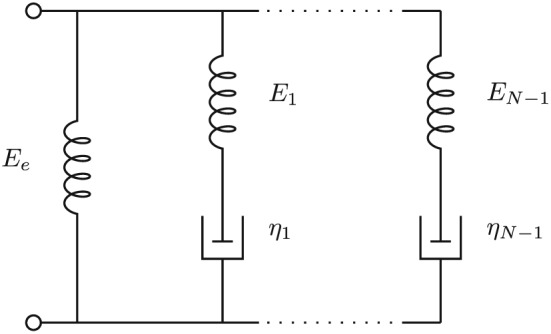
Maxwell–Wiechert model, consisting of parallel networks of spring–damper models

A physically realizable model is obtained if *G*(*t*) is modeled by a parallel network of *N*−1 pairs of springs and dashpots in series where the spring constants are *E*_*n*_ and the viscosities of the dashpots are *η*_*n*_=*τ*_*n*_ *E*_*n*_. Furthermore, both the spring constants and the viscosities are non‐negative. This is the Maxwell–Wiechert model of Figure 1. The additional left‐hand spring leads to the constant *G*_e_=*E*_e_, the equilibrium modulus, and if there is a dashpot directly across the terminals (e.g. if *E*_1_=0) then the response will have an impulse at time zero as well, given by *G*_−_. This is the case in the Kelvin–Voigt model, which will soon be discussed.

The model of Equation 2 can also be expressed as a convolution, as in Equation 1. However, here we are interested in that subset of convolution kernels that can be characterized as fading memory kernels and not all the coefficient sets of Equation 2 will lead to such a model. Therefore the linear differential equation is the most general model. Also, a model described as a relaxation spectrum (Equation (3)) can always be described with a linear differential equation, but not vice versa. In addition, a relaxation spectrum model, Equation 3, always results in a fading memory model, as it consists of sums of positively weighted falling exponentials.

The frequency‐domain equivalent of Equation 2 is also useful:

(4) $$E(\omega )=\frac{\sigma (\omega )}{\epsilon (\omega )}=\frac{E_{e}+\sum_{k=1}^{q}b_{k}{\left(i\omega \right)}^{k}}{1+\sum_{k=1}^{p}a_{k}{\left(i\omega \right)}^{k}}$$

The dynamic modulus, E(ω), is often called G(ω) in elastography, but, since G means the relaxation modulus in this article, E is used instead.

### 2.2 Elementary linear viscoelastic models

**Figure 2:**
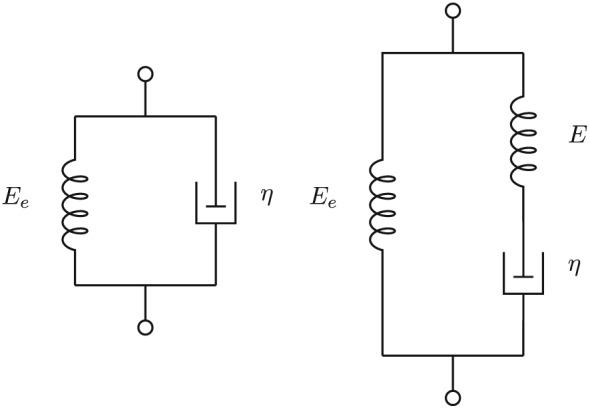
Kelvin–Voigt (left) and Zener models. As shown in this article, the three‐component Zener model also models the shear wave in the Biot poroelastic model

The two simplest viscoelastic models are the Kelvin–Voigt and the Zener models, as shown in Figure 2. The Kelvin–Voigt model is found by just keeping E_e_ and η_1_ in Figure 1. The linear differential equation is

(5) $$\sigma (t)=E_{e}\epsilon (t)+\eta \frac{\partial \epsilon (t)}{\partial t}$$

and the relaxation modulus is

(6) $$G(t)=E_{e}+\eta \delta (t)$$

Thus p=0, q=1, a_1_=0, and b_1_=η in Equations 2 and 4. Further, G_e_=E_e_, G_−_=η, and G_τ_(t)=0 in Equation 3. The dynamic modulus is

(7) $$E(\omega )=E_{e}+i\omega \eta =E_{e}(1+i\omega \tau_{\epsilon })$$

where τ_ϵ_=η/E_e_. The even simpler elastic model, which is often the reference in elastography, as noted in the Introduction, is obtained by setting the viscosity, η, to 0.

The Zener model adds one more term to the linear differential equation of the Kelvin–Voigt model:

(8) $$\sigma (t)+a_{1}\frac{\partial \sigma (t)}{\partial t}=E_{e}\epsilon (t)+\eta \frac{\partial \epsilon (t)}{\partial t}$$

and the relaxation modulus is

(9) $$G(t)=E_{e}+E_{e}\left(\frac{\tau_{\epsilon }}{\tau_{\sigma }}-1\right)e^{-t/\tau_{\sigma }}$$

Compared with the Kelvin–Voigt model, the effect of the second spring is to ‘soften’ the impulse in Equation 6 into a falling exponential. In this model, *p*=*q*=1 and *b*_1_=*η* in Equations 2 and 4. The time constants are *τ*_*ϵ*_, as in the Kelvin–Voigt model, and *τ*_*σ*_=*a*_1_. The parameters of the model relate to the physical parameters of Figure 1 as follows:

(10) $$\tau_{\sigma }=\eta /E\leq \tau_{\epsilon }=\eta /E^{\prime },\;\frac{1}{E^{\prime }}=\frac{1}{E_{e}}+\frac{1}{E}$$

For this model, the dynamic modulus is

(11) $$E(\omega )=E_{e}\frac{1+i\omega \tau_{\epsilon }}{1+i\omega \tau_{\sigma }}$$

The addition of the extra spring leads to a frequency‐dependent denominator in the dynamic modulus, which gives more degrees of freedom in fitting this model to data.

The dynamic modulus can also be included in a dispersion relation when propagating waves are involved. In Equations 13, 14, 15 of Holm and Näsholm,^16^ it is shown that the complex wave number, *k*, in that case is

(12) $${\left(\frac{k}{\omega }\right)}^{2}=\rho \kappa (\omega )=\frac{\rho }{E(\omega )}$$

where *ρ* is the density and *κ*(*ω*) is the dynamic compliance or compressibility, the inverse of the dynamic modulus. This result is important in the subsequent analysis of the poroelastic model. Note that the roles of *τ*_*ϵ*_ and *τ*_*σ*_ are reversed here, compared with the work of Holm and Näsholm.^16^ Here the convention of Mainardi^13^ is followed instead.

A slightly rewritten version of the compliance based on factoring of Equation 11 will be needed when analyzing the poroelastic model:

(13) $$\kappa (\omega )=\frac{1}{E(\omega )}=\frac{1}{E_{e}}\frac{1+\omega^{2}\tau_{\epsilon }\tau_{\sigma }-i\omega (\tau_{\epsilon }-\tau_{\sigma })}{1+\omega^{2}\tau_{\epsilon }^{2}}$$

Judging from Figure 1, a Zener model is characterized by two springs and a damper. These three parameters may be expressed in different ways and one common way is via the low‐frequency asymptote of the propagation speed, *c*_0_, and the two crossover frequencies, *ω*_*ϵ*_=1/*τ*_*ϵ*_ and *ω*_*σ*_=1/*τ*_*σ*_. The two frequencies express approximately where the phase velocity starts rising and where it approaches its asymptotic value,^16^ $c_{\infty }=c_{0}\sqrt{\omega_{\sigma }/\omega_{\epsilon }}$. Taking into consideration that $c_{0}^{2}=E_{e}/\rho$ and thus depends on both the shear modulus and the density, then a Zener medium will depend on four independent parameters: *E*_e_, *ρ*, *ω*_*ϵ*_, and *ω*_*σ*_.

### 2.3 Time‐ and frequency‐spectral functions

The continuous generalization of Equation 3 is given by(13, 17, 18)

(14) $$G_{\tau }(t)=b\int_{0}^{\infty }R_{\sigma }(\tau )e^{-t/\tau }\;d\tau$$

where *b*=*G*_*τ*_(0) is a non‐negative constant, which for the Zener model is *b*=*E*(*τ*_*ϵ*_/*τ*_*σ*_−1). 18 *R*_*σ*_(*τ*) is a non‐negative relaxation spectrum.

There is a corresponding frequency‐spectral function, which is

(15) $$S_{\sigma }(\Omega )=b\frac{R_{\sigma }(1/\Omega )}{\Omega^{2}}$$

By substituting Ω=1/*τ* so that dΩ=−d*τ*/*τ* ^2^, this gives

(16) $$G_{\tau }(t)=\int_{0}^{\infty }S_{\sigma }(\Omega )e^{-\Omega t}\;d\Omega$$

**Figure 3:**
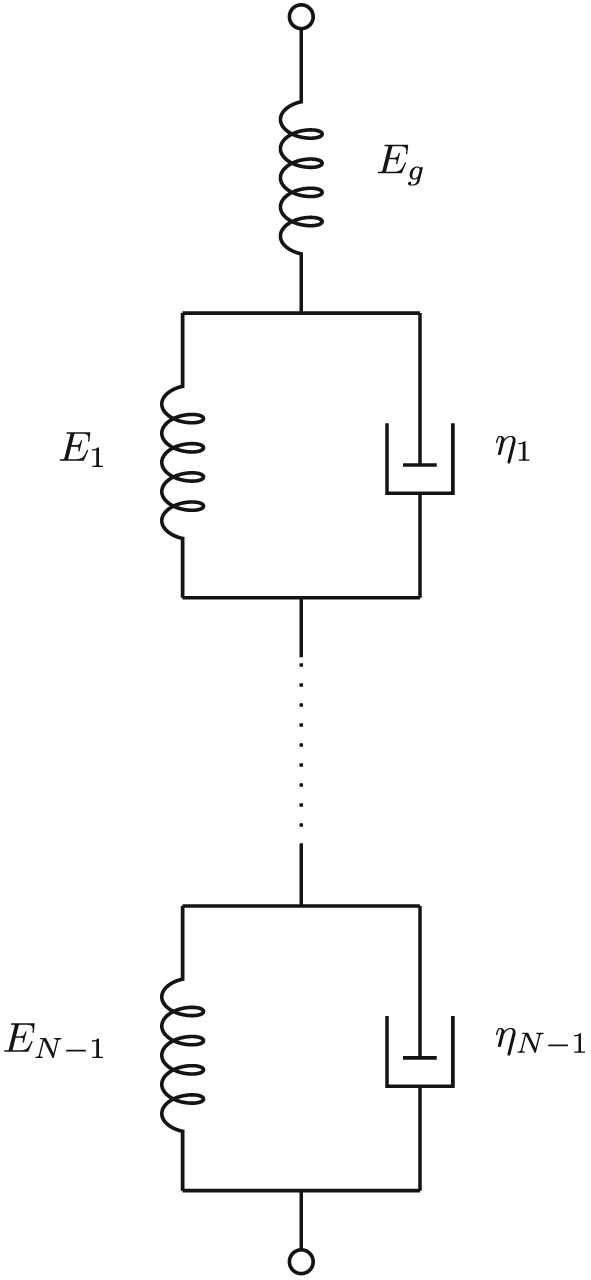
Kelvin model consisting of series networks of spring–damper models. It is the conjugate of the Maxwell–Wiechert model of Figure 1

The relaxation modulus is, as noted, the stress response of the system to a step excitation in strain. The relaxation spectrum could also have been expressed with the creep response, which is the strain response to a step excitation in strain. It is denoted by *J*(*t*) and plays the same role as *G*(*t*) in Equation 1 if *σ*(*t*) and *ϵ*(*t*) are interchanged. The time‐spectral function is

(17) $$J_{\tau }(t)=a\int_{0}^{\infty }R_{\epsilon }(\tau )(1-e^{-t/\tau })\;d\tau$$

where $a=J_{\tau }(\infty )$ is a non‐negative constant, which for the Zener model is *a*=(1/*E*)(1−*τ*_*σ*_/*τ*_*ϵ*_). 18 This corresponds to a decomposition into a sum of elementary models in series, as shown in Figure 3. This is the conjugate of the Maxwell–Wiechert model of Figure 1, i.e. a different configuration of springs and dashpots that has the same characteristics.^19^ It follows that the conjugate of the Zener model in the right‐hand part of Figure 2 is also the top three elements of Figure 3.

The frequency‐spectral function for the creep is found by a transformation analogous to that for relaxation:

(18) $$S_{\epsilon }(\Omega )=a\frac{R_{\epsilon }(1/\Omega )}{\Omega^{2}}$$

and the frequency‐spectral function is

(19) $$J(t)=\int_{0}^{\infty }S_{\epsilon }(\Omega )(1-e^{-\Omega t})d\Omega$$

This frequency spectral function will be used to find the spring–damper equivalent of the fractional models.

## 3 FRACTIONAL MODELS

Both the Kelvin–Voigt model and the Zener model can be generalized by introducing non‐integer, fractional derivatives of order *α* in the constitutive equation (Equation (2)). For the Zener model, this is

(20) $$\sigma (t)+\tau_{\sigma }^{\alpha }\frac{\partial^{\alpha }\sigma (t)}{\partial t^{\beta }}=E\left[\epsilon (t)+\tau_{\epsilon }^{\alpha }\frac{\partial^{\alpha }\epsilon (t)}{\partial t^{\alpha }}\right]$$

**Figure 4:**
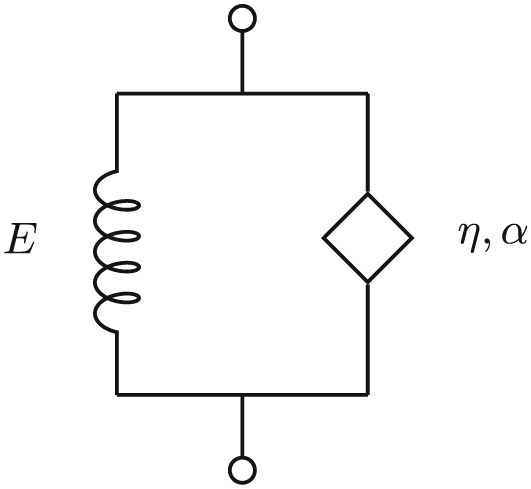
Fractional Kelvin–Voigt model with spring characterized by the shear modulus, E, and spring‐pot given by shear viscosity, η, and fractional order, α

The dynamic modulus is now

(21) $$E(\omega )=E_{e}\frac{1+{\left(i\omega \tau_{\epsilon }\right)}^{\alpha }}{1+{\left(i\omega \tau_{\sigma }\right)}^{\alpha }}$$

while, in the simpler Kelvin–Voigt model shown in Figure 4, the constant *τ*_*σ*_=0:

(22) $$\sigma (t)=E\left[\epsilon (t)+\tau^{\alpha }\frac{\partial^{\alpha }}{\partial t^{\alpha }}\epsilon (t)\right]$$

The dynamic modulus is

(23) $$E(\omega )=E_{e}\left(1+{\left(i\omega \tau_{\epsilon }\right)}^{\alpha }\right)$$

In the limit as the frequency and/or viscosity becomes very large, the dynamic modulus approaches $E(\omega )\to {\left(i\omega \eta \right)}^{\alpha }$. This is equivalent to the case where the spring of Figure 4 can be neglected. That seems often to be the case for elastography data.(20, 21) Publications report observed values for the exponent, *α*, in the range 0.06–0.27 for ultrasound elastography in liver and prostate and 0.26–0.3 for MR elastography of liver. Similarly, Ormachea et al^22^ demonstrated that such low values of *α* are consistent between different ultrasound elastography methods in liver.

The relaxation modulus of the fractional Kelvin–Voigt model is expressed by a power‐law function of time, while for the fractional Zener model it follows a Mittag–Leffler function. That function is a generalization of the exponential function with power‐law behavior for large time. The creep compliance of both models also follows a Mittag–Leffler function. That means that the creep time‐ and frequency‐spectral functions of the two models will also be similar.

### 3.1 Long‐tailed time‐ and frequency‐spectral function

In previous works,(13, 17, 18) it has been shown that, for the fractional Zener model, the creep time‐spectral function of Equation 17 is

(24) $$R_{\epsilon }(\tau )=\frac{1}{\pi \tau }\frac{\sin\alpha \pi }{{\left(\tau /\tau_{\epsilon }\right)}^{\alpha }+{\left(\tau /\tau_{\epsilon }\right)}^{-\alpha }+2\cos\alpha \pi }$$

It was plotted by Caputo and Mainardi(13, 18) with linear axes and is a decreasing function of *τ* for small *α*, with a more and more pronounced peak as *α* approaches 1. For the non‐fractional case, *α*=1, it is a delta function at *τ*/*τ*_*ϵ*_=1, showing that it is equivalent to a single relaxation process in that case.

**Figure 5:**
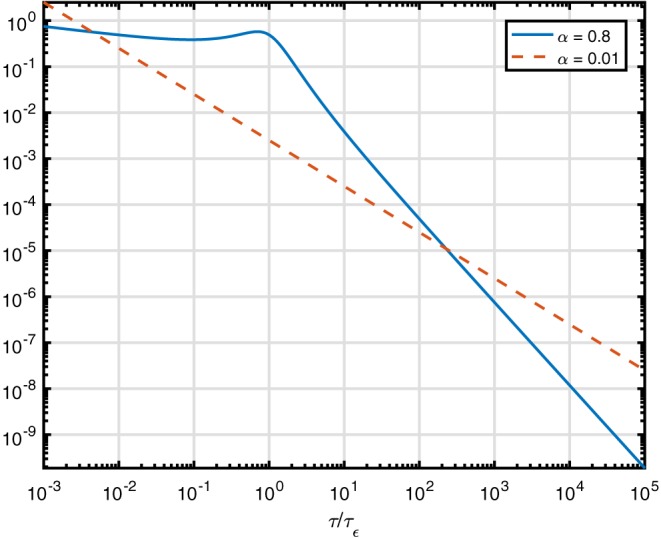
Time‐spectral function for the fractional Zener and Kelvin–Voigt models. The solid line denotes α=0.8, the dashed line α=0.01

Here the function is plotted on a log–log scale in Figure 5. That brings out the properties of this particular function in a different way than if it were plotted in a linear plot. The log–log plot fixes attention on the asymptotes, rather than the peak, and they are

(25) $$R_{\epsilon }(\tau )\propto \left\{\begin{array}{cc} \tau^{\alpha -1} & \text{for}\;\tau /\tau_{\epsilon }\ll 1\\ \tau^{-\alpha -1} & \text{for}\;\tau /\tau_{\epsilon }\gg 1 \end{array}\right.$$

The distribution of elementary spring–damper models in the medium, as given in Figures 1 and 3, has different asymptotes for small and large time and both of them are power laws. As long as the exponent of the high‐frequency tail falls off more slowly than *τ* ^−2^, i.e. for *α*<1, the variance of the distribution will not exist. Such long‐tailed distributions are scale‐invariant.

In Mainardi's works,(13, 23) the frequency‐spectral function is also given. It can be found from Equation 18 using the normalizing constant $a=J_{\tau }(\infty )=(1/E)(1-{\left(\tau_{\sigma }/\tau_{\epsilon }\right)}^{\alpha })$ for the fractional Zener model. The creep frequency‐spectral function of Equation 19 is then

(26) $$S_{\epsilon }(\Omega )=\frac{1}{\pi E}\frac{(\tau_{\epsilon }^{\alpha }-\tau_{\sigma }^{\alpha })\Omega^{\alpha -1}\sin\alpha \pi }{{\left(\Omega \tau_{\epsilon }\right)}^{2\alpha }+1+2{\left(\Omega \tau_{\epsilon }\right)}^{\alpha }\cos\alpha \pi }$$

This result was given by Näsholm and Holm^24^ in the form above. There, it was derived with a starting point in the multiple relaxation theory of Nachman et al.^25^ It should be noted that the frequency‐spectral function is exactly the same as the time‐spectral function, except for a scaling factor.

**Figure 6:**
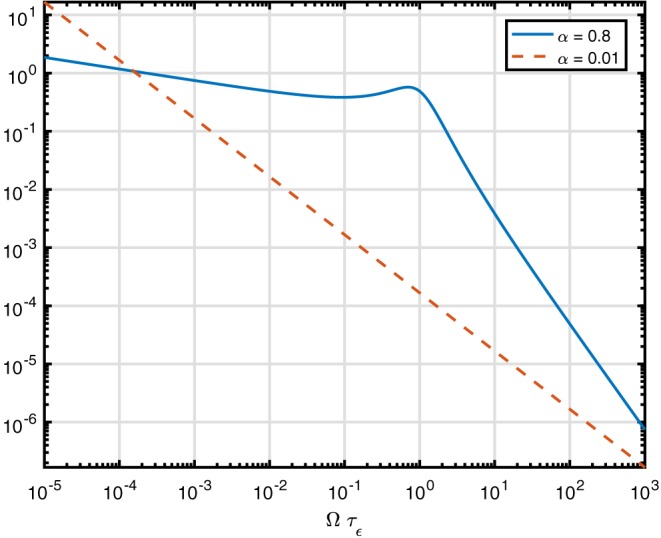
Frequency‐spectral function for the fractional Zener model for τ_σ_=1000τ_ϵ_. The solid line denotes α=0.8 as in Näsholm and Holm,^24^ the dashed line α=0.01. It is also valid for the Kelvin–Voigt model, except for an amplitude scaling

The plot for *α*=0.8 and *α*=0.01 in Figure 6 shares many properties with the plot of the time‐spectral function. The asymptotes are also very similar^26^:

(27) $$S_{\epsilon }(\Omega )\propto \left\{\begin{array}{cc} \Omega^{\alpha -1} & \text{for}\;\Omega \tau_{\epsilon }\ll 1\\ \Omega^{-\alpha -1} & \text{for}\;\Omega \tau_{\epsilon }\gg 1 \end{array}\right.$$

Interestingly, the relaxation spectrum approaches a single power law for the limiting case α→0, where both the low‐ and high‐frequency parts of the relaxation spectrum will approach Ω^−1^. See the dash–dotted line for *α*=*ϵ*=0.01 in Figure 6, with asymptotes Ω^−1+*ϵ*^ for low frequencies and Ω^−1−*ϵ*^ for high frequencies. As shown by Holm and Näsholm,^16^ α→0 results in attenuation which approaches *f* ^1^. Such an attenuation law was analyzed by Pauly and Schwan,^27^ who also found the Ω^−1^ weighting. The result of Equation 26 can therefore be viewed as a generalization of that result to power laws other than 1.

### 3.2 Relationship with fractals

Much effort has been expended on finding the relationship between fractional models and fractal geometries, beyond the semantic similarity. A general connection has not been found, but a geometrical and physical interpretation of fractional integration and differentiation was given by Podlubny. 28

There are also studies that are more specific to wave propagation and attenuation. The results are then often for the alternative attenuation mechanism of scattering rather than absorption. This has been shown theoretically by Fouque et al^29^ and Garnier and Sølna.^30^ Similarly, Lambert et al^31^ showed both theoretically and experimentally that a fractal distribution of scatterers leads to attenuation that varies with frequency according to a power law.

Likewise, Posnansky et al^32^ and Guo et al^33^ studied hierarchical fractal models and built fractal phantoms using tangled paper strips in an agarose gel to emulate polymer chains embedded in an elastic matrix. The fractal dimension was varied and it was found experimentally that, as the fractal dimension increased, so did the power‐law exponent in the range *α*=0.15−0.24.

The microchannel model of Parker^34^ is also a hierarchical model. It is a Maxwell–Wiechert model as in Figure 1 (with *E*_e_=0) and is based on the flow of viscous fluids through an extensive network of tissue microchannels. Each microchannel is modelled as a series connection of a spring and a damper. A weighting of each contribution, similar to Equation 24, was proposed. A dynamic modulus proportional to $E(\omega )\propto \omega^{\alpha }$ results in a time‐spectral function that is weighted with *τ* ^−*α*−1^, which is the same result as the asymptote of Equation 25 for large time (lower line), i.e. the most important region. Such a power‐law dynamic modulus can be modeled with Equations 21 and 23 under certain conditions for the parameters, at least over a limited frequency range, as mentioned in the discussion following Equation 23.

### 3.3 Relationship with hysteresis

The results found here have some resemblance to those derived from hysteresis. Hysteresis is a hypothetical loss element with a constant phase lag between stress and strain at all frequencies,^35^ resulting in attenuation that increases linearly with frequency.^36^ The dynamic modulus for hysteresis is

(28) E(ω)=K+iH

where *K* and *H* are constants. The model leads to a noncausal model and therefore^35^ is concerned with making a band‐limited approximation, where the constants are allowed to vary with frequency, *K*(*ω*) and *H*(*ω*), in order to ensure causality.

By comparing the dynamic moduli, Equations 28 and 23, it is evident that hysteresis can be viewed as the limiting case as α→0 for the fractional Kelvin–Voigt model. Thus an alternative way to ensure causality is to employ a fractional Kelvin–Voigt model with parameter *α*=*ϵ*=0^+^.

Hysteresis is particularly interesting in the context of fractional operators, because the result for the limiting case α→0 of the previous section is similar to that recently found for hysteresis by Parker.^37^ In Parker's work, it was shown that the hysteresis model is the same as a sum of relaxation processes weighted with a long‐tailed power law Ω^−1+*ϵ*^. This parallels our discussion of the asymptotic result of Equation 27 for *α*=*ϵ* and Parker's result can be interpreted as a special case of Equation 27.

## 4 POROELASTIC MODEL

The Biot poroelastic model deals with a saturated porous medium with a solid phase and a fluid phase. Wave propagation in such a medium is described by a set of coupled vector wave equations, as given in Equation 4.2 of Biot.^11^ The variables are the displacement vectors **u** and **U** for the displacement in the solid and the fluid, respectively. That article assumes that, as the fluid moves in the pores, the flow is laminar and that losses are given by Darcy's law and are proportional to the relative velocity, *∂*(**u**−**U**)/*∂* *t*. The theory predicts three solutions: two compressional waves and one shear wave. In a biological porous medium like cancellous bone (bone with a low volume fraction of solid, less than 70%), all three waves have been detected.^38^

This model is much more complex than the viscoelastic models of the previous sections. It also seems more appropriate for a complex medium like brain, liver, or bone. Here, a surprising exact relationship between the poroelastic and viscoelastic models will be shown for the shear‐wave solution.

### 4.1 Biot's original formulation

As elastography is only concerned with shear waves, we restrict the analysis here to that mode. The following dispersion relation was derived by Biot^11^ (Equations 7.5–7.6 therein):

(29) $${\left(\frac{k}{\omega }\right)}^{2}=\rho (\kappa_{r}-i\kappa_{i}),$$

where

(30) $$\begin{array}{ccc} \kappa_{r} & =\frac{1}{\mu }\frac{1+\gamma_{22}\frac{\gamma_{11}\gamma_{22}-\gamma_{12}^{2}}{{\left(\gamma_{12}+\gamma_{22}\right)}^{2}}{\left(\frac{f}{f_{c}}\right)}^{2}}{1+{\left(\frac{\gamma_{22}}{\gamma_{12}+\gamma_{22}}\right)}^{2}{\left(\frac{f}{f_{c}}\right)}^{2}} & \\ \kappa_{i} & =\frac{1}{\mu }\frac{f}{f_{c}}\frac{\gamma_{12}+\gamma_{22}}{1+{\left(\frac{\gamma_{22}}{\gamma_{12}+\gamma_{22}}\right)}^{2}{\left(\frac{f}{f_{c}}\right)}^{2}} \end{array}$$

This expression depends on three normalized densities:

(31) $$\gamma_{11}=\frac{\rho_{11}}{\rho },\;\gamma_{22}=\frac{\rho_{22}}{\rho },\;\gamma_{12}=\frac{\rho_{12}}{\rho }$$

and the aggregate or composite density, which is

(32) $$\rho =\phi \rho_{f}+(1-\phi )\rho_{s}.$$

These formulae depend on the porosity, *ϕ*, and the fluid and solid densities. The parameter *ρ*_12_ represents a negative mass coupling density between fluid and solid, *ρ*_11_ represents the total effective mass of the solid moving in the fluid, and *ρ*_22_ is the total effective mass of the fluid. There is also a characteristic frequency:

(33) $$f_{c}=\frac{b}{2\pi (\rho_{12}+\rho_{22})},\;b=\frac{\eta \phi^{2}}{B}$$

where *η* is the fluid viscosity and *B* is the permeability. The effective characteristic frequency of Equation 30 is, however, a lower frequency:

(34) $$f_{c}^{\prime }=f_{c}\frac{\gamma_{12}+\gamma_{22}}{\gamma_{22}}=\frac{1}{2\pi }\frac{\eta \phi }{B\alpha \rho_{f}}$$

since *ρ*_22_=*α* *ϕ* *ρ*_f_,^39^ where *α* is a structure constant related to tortuosity.

Comparison with Equations 12 and 13 shows that Equations 29 and 30 are exactly the same as those of the Zener model. This is a remarkable result, which shows that, for shear waves, the poroelastic model is also a linear viscoelastic model. What distinguishes it from ordinary linear viscoelastic models is that it provides a sophisticated way of determining the parameters from physical considerations.

### 4.2 Biot–Stoll formulation

The parameters of the original Biot theory are often considered to be hard to find in practice and the theory has been rewritten in terms of the relative displacement between fluid and solid, **u** **−** **U**, or the volume of fluid that has flowed in or out of an element, *ζ*=*ϕ*∇(**u**−**U**), in combination with the solid displacement **u**. The material parameters are then transformed to a new set of parameters.

In that case, Hovem's^40^ Equation 16.77 gives the shear dispersion of the low‐frequency Biot model. It can also be found from Equation 12 of Stoll^12^:

(35) $${\left(\frac{k}{\omega }\right)}^{2}=\frac{\rho }{\mu }\frac{(\rho_{c}-\frac{\rho_{f}^{2}}{\rho })-i\frac{\eta }{\omega B}}{\rho_{c}-i\frac{\eta }{\omega B}}=\frac{\rho }{\mu }\frac{1+i\omega \left(\rho_{c}-\frac{\rho_{f}^{2}}{\rho }\right)\frac{B}{\eta }}{1+i\omega \frac{\rho_{c}B}{\eta }}$$

where the sign of *ω* has been reversed compared with the original articles, due to a different definition of the Fourier transform here (see also Appendix A of Holm and Näsholm^41^). The mass coupling density is

(36) $$\rho_{c}=\alpha \rho_{f}/\phi$$

where *α* is the tortuosity. Comparing Equation 35 with Equation 12 shows that the dynamic modulus is

(37) $$E(\omega )=\rho {\left(\frac{\omega }{k}\right)}^{2}=\mu \frac{1+i\omega \frac{\rho_{c}B}{\eta }}{1+i\omega \left(\rho_{c}-\frac{\rho_{f}^{2}}{\rho }\right)\frac{B}{\eta }}$$

As expected, this is also equivalent to that of a Zener model, as can be seen by comparison with Equation 11. The constants when *ω*_*ϵ*_=1/*τ*_*ϵ*_ and *ω*_*σ*_=1/*τ*_*σ*_ are

(38) $$E_{e}=\mu_{r},\;\omega_{\epsilon }=\frac{\eta }{\rho_{c}B},\;\omega_{\sigma }=\frac{\omega_{\epsilon }}{1-\frac{\rho_{f}^{2}}{\rho \rho_{c}}}\geq \omega_{\epsilon },\;c_{0}^{2}=\frac{\mu }{\rho }$$

Insertion of the expression for the mass coupling density, Equation 36, in the equation for the effective characteristic frequency, Equation 34, shows that this frequency is also the same as *ω*_*ϵ*_/(2*π*).

### 4.3 Redundant parameters in the Biot shear‐wave model

The low‐frequency Biot model is characterized by the ten parameters shown in Table 1. Seven of them affect shear‐wave propagation, as indicated in the right‐hand column, but several of them are connected, as Equation 38 indicates.

**Table 1:** Biot–Stoll parameters and their effect on the shear wave. The pore radius parameter is not included, as the flow in the pores is assumed to be laminar. The right‐hand column shows whether the parameter affects the shear wave or not

**Biot model parameters**			Shear?
**Bulk properties:**			
*ϕ*	[0…1]	Porosity	Y
*ρ*_s_	[kg/m^3^]	Solid density	Y
*ρ*_f_	[kg/m^3^]	Fluid density	Y
*K*_s_	[Pa]	Bulk modulus, solid	N
*K*_f_	[Pa]	Bulk modulus, fluid	N
**Fluid parameters:**			
*η*	[Pa s]	Viscosity	Y
*B*	[m^2^]	Permeability	Y
*α*	[1…3]	Tortuosity	Y
**Rigid frame response parameters:**			
*K*_r_	[Pa]	Bulk modulus	N
*μ*_r_	[Pa]	Shear modulus	Y

The parameter *ω*_*ϵ*_ depends on the mass coupling density *ρ*_c_. In addition it depends on the ratio of the viscosity, *η*, and the permeability, *B*. They do not influence any of the other parameters, *ω*_*σ*_ and *c*_0_, so it is clear that it is their ratio that matters. Likewise, the sound velocity, *c*_0_, depends on the ratio of the shear modulus, *μ*_r_, and the aggregate density, *ρ*.

Furthermore, for the third parameter, *ω*_*σ*_, the denominator in the expression is usually larger than 0.8, so the ratio of *ω*_*σ*_ and *ω*_*ϵ*_ is not very sensitive to changes in the densities *ρ*, *ρ*_c_, or *ρ*_f_. That means that neither is it very sensitive to changes in tortuosity and it will mainly be *ω*_*ϵ*_ that depends on *α* in the ratio *η*/(*α* *B*).

In this way, three of the seven parameters can be said to be redundant and the seven parameters from Table 1 (*η*, *B*, *α*, *ρ*_f_, *ρ*_s_, *ϕ*, *μ*_r_) may be reduced to four by combining the first three into one, *η*/(*α* *B*). Alternatively, the four parameters may be stated as *η*/*α*, *ρ*_c_, *ρ*, and *μ*_r_. In case one wants to compute *ω*_*σ*_ exactly, a fifth parameter, *ρ*_f_, also needs to be included.

In this way, the number of parameters in the Biot shear model with some approximation is four, as in the Zener medium model, and five in the exact case.

### 4.4 Extension to poroviscoelasticity

In the sediment acoustics field, the Biot model has been amended in order to extend the model from a rigid porous frame, like a porous rock or bone, to one where the grains are allowed to move. This is a model for a saturated sediment and it is not unlikely that it may be more appropriate for tissue than the rigid frame implied in the Biot model.

In this case, viscosity is introduced in the solid frame in addition to the flowing liquid. The model is called the Biot squirt flow and viscous drag (BICSQS) model.^42^ This viscosity is added by allowing a relaxation model for the shear modulus, thus allowing for a frequency‐dependent complex shear modulus or dynamic shear modulus, described by a characteristic frequency *ω*_*μ*_:

(39) $$\mu^{\prime }=\mu \left(1+i\frac{\omega }{\omega_{\mu }}\right)$$

When the relaxation is included in Equations 37 and 11, the result is

(40) $$E(\omega )=\mu \frac{\left(1+i\omega /\omega_{\mu })(1+i\omega /\omega_{\epsilon }\right)}{1+i\omega /\omega_{\sigma }}$$

**Figure 7:**
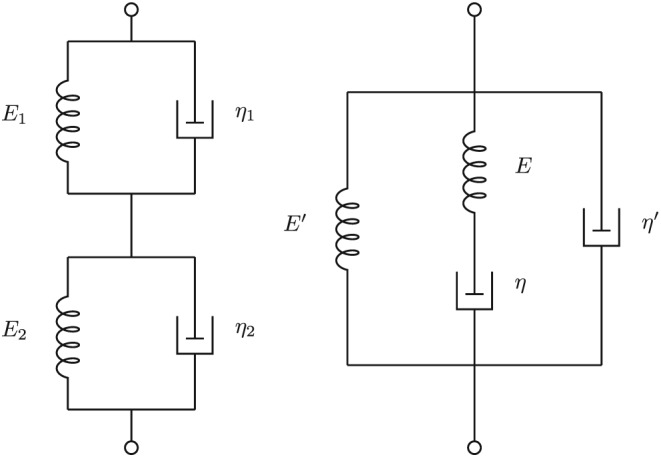
The equivalent to the shear‐wave poroviscoelastic model, called the non‐standard four‐parameter model by Tschoegl^19^ (left) with its conjugate to the right

In the limit, as the frequency tends to zero the dynamic modulus approaches *E*(0)=*μ* and as the frequency approaches infinity it approaches E(∞)=∞. This model is called the non‐standard four‐parameter model.^19^ Its spring–damper realization is shown in Figure 7, with the equivalent conjugate model to the right. Therefore, even in this case an equivalent viscoelastic model may be found.

This model has been further developed to cover water‐saturated sand as well. In that case, there is also coupling between the relaxation model for the bulk modulus and that for the shear modulus; see Equation 15 of Chotiros and Isakson.^43^ The coupling term is a complex function of frequency that involves Bessel functions, but it can often be approximated with a simpler polynomial expression.^44^ In that case, the model may also be expressed by springs and dampers, but an even more complex system than that of Figure 7. However, for biological tissue, the simpler model of Equation 39 should be adequate.

## 5 DISCUSSION AND CONCLUSION

The concept of time‐ and frequency‐spectral decompositions of viscoelastic systems has been developed and applied to the fractional Zener and Kelvin–Voigt models. This summation of multiple elementary models is an idea that is independent of the fractional models. In fact, it is the idea behind the relaxation‐spectrum models of Kelvin and Wiechert dating from 1888 and 1893, respectively.^15^ In addition to the coverage by Mainardi^13^, Tschoegl's book^19^ devotes several sections to it (Chapters 3.5–3.6). Similar ideas of model fitting have also been used in biomechanics and elastography. In the white matter model of Cheng and Bilston,^45^ the shear relaxation modulus was, for instance, modeled with three very slow relaxation terms (0.01 Hz and slower) and a similar model with two terms was used by Caenen et al. 46

The idea behind the particular weightings implied in the time‐spectral and frequency‐spectral functions found here is, however, to make the sum approximate the behaviour of the fractional models, i.e. in terms of power laws in the frequency domain. Therefore it parallels the modeling of arbitrary power‐law attenuation in medical ultrasound over a limited frequency range with a few terms by Yang and Cleveland. 47 The surprising result is that the weighting has a long‐tailed distribution, with different power laws for low and high arguments. Here it characterizes the distribution of individual relaxation processes, in both time and frequency domains.

The poroelastic model depends on ten independent parameters, of which seven influence the shear‐wave solution, and seems to build on an entirely different foundation from viscoelastic models. Despite this, it has been shown to be equivalent to a Zener model. This was done by comparing dispersion relations and, not unsurprisingly, the result is the same whether the dispersion relations from the original Biot theory or those from the Biot–Stoll theory are analyzed. This result is an extension of the work of Bardet,^48^ where it was shown that, in the low‐loss/low‐frequency approximation, an equivalent can be found between the poroelastic model and a Kelvin–Voigt model. This was done by comparing approximate expressions for the velocity and attenuation. As the Zener model in the low‐loss/low‐frequency case is equivalent to the Kelvin–Voigt model,^16^ the result found here also agrees with that result.

It is also shown that the seven parameters of the poroelastic model can be reduced to five, and even four as in the Zener model, if a small approximation is allowed. When the Biot model is extended to include viscosity in the frame, as in the work of Chotiros and Isakson,^42^ an extra damper has to be added to the Zener model, making it into what is called the non‐standard four‐parameter model. 19
